# Chemical composition of the giant red sea cucumber, Parastichopus californicus, commercially harvested in Alaska

**DOI:** 10.1002/fsn3.12

**Published:** 2013-01-08

**Authors:** Peter J Bechtel, Alexandra CM Oliveira, Necla Demir, Scott Smiley

**Affiliations:** 1 USDA-ARS, SARU, Kodiak Seafood and Marine Science Center, 118 Trident WayKodiak, AK, 99615; 2 Kodiak Seafood and Marine Science Center, University of Alaska Fairbanks, 118 Trident WayKodiak, AK, 99615; 3 American University in Cairo, Department of Chemistry, AUC Avenue P.O. Box 74New Cairo, Egypt, 11385

**Keywords:** Echinoderms, giant red sea cucumbers, marine fatty acids, Parastichopus californicus, sea cucumbers

## Abstract

Giant red sea cucumbers, *Parastichopus californicus*, are commercially harvested in the U.S. Pacific Northwest; however, the nutritional and chemical properties of its edible muscle bands and body wall have not been fully elucidated. In particular are the fatty acid profiles of *P. californicus* tissues, which have not been documented. Sea cucumbers were delivered live and muscle bands and body wall freeze dried, vacuum packed, and stored at –30°C until analyzed. Proximate composition of freeze-dried tissues varied greatly with muscle bands being composed of 68% protein, 12% ash, 9% carbohydrate, and 5% lipids, while the body wall was composed of 47% protein, 26% ash, 15% carbohydrate, and 8% lipids. The hydroxyproline, proline, and glycine contents of the body wall were much higher than those in muscle bands, consistent with the larger amount of connective tissue. Calcium, magnesium, sodium, and iron contents were higher in the body wall than those in muscle bands, whereas the opposite was observed for zinc content. Total long-chain *n*-3 fatty acid contents were 19% and 32% of total fatty acids in body wall and muscle bands, respectively. Muscle bands had higher content of eicosapentaenoic acid (20:5*n*-3) than body wall at 22.6% and 12.3%, respectively. High content of arachidonic acid (20:4*n*-6) was recorded in both body wall (7.1%) and muscle bands (9.9%). Overall, the fatty acid profiles of body wall and muscle bands of *P. californicus* resemble those described for other species; however, the distribution and occurrence of certain fatty acids is unique to *P. californicus*, being representative of the fatty acid composition of temperate-polar marine organisms. The chemical characterization of freeze-dried edible tissues from *P. californicus* demonstrated that these products have valuable nutritional properties. The body wall, a food product of lower market value than muscle bands, could be better utilized for nutraceutical and pharmaceutical applications.

## Introduction

Sea cucumbers are Echinoderms, a phylum also containing starfish and sea urchins. The class Holothuroidea is taxonomically diverse and contains about 1500 species (Smiley 1994). Some of the unique life-history traits of holothurians are great longevity, low or infrequent recruitment, and density-dependent reproductive success (Purcell 2010). Commercial sea cucumber fisheries focus on a number of species belonging to the order Aspidochirotida and several species in the order Dendrochirotida. The latest estimates for global annual catch of sea cucumbers are on the order of 100,000 t (Purcell 2010). An ever-increasing market demand for sea cucumbers worldwide, combined to rampant exploitation and inadequate fishery management of sea cucumber stocks in certain parts of the world, make these species especially vulnerable to overfishing (Purcell 2010).

According to Toral-Granada et al. (2008), the commercial species of sea cucumbers found in the North Atlantic and North Pacific are *Cucumaria frondosa*, *Cucumaria japonica*, *Parastichopus californicus,* and *Parastichopus parvimensis*. Commercial sea cucumber fisheries take place in U.S. waters off the Pacific coast states of California, Oregon, Washington, and Alaska, and on the Atlantic off the coast of Maine. Two species are commercially fished along the Pacific coast, but the giant red sea cucumber, *Parastichopus californicus*, is the only commercially harvested sea cucumber in Alaska (Alaska Department of Fish and Game [ADFG] 2012). Alaska's largest sea cucumber fishery occurs in Southeast Alaska, while smaller fisheries occur in the Kodiak Archipelago and along the Alaskan Peninsula (Alaska Department of Fish and Game 2012). Most of the sea cucumbers harvested by divers in Alaska (300,000–400,000 kg/year) and along the North American Pacific coast are exported to Hong Kong, Taiwan, Mainland China, and Korea (Alaska Department of Fish and Game 2012), although there are small ethnic markets in the U.S. and Canada.

Sea cucumbers have been consumed in Asian countries for centuries for their dietary and curative properties (Toral-Granada et al. 2008). Many Asians believe sea cucumbers should be eaten to treat ailments such as cancer and arthritis, as well as intestinal and urinary dysfunctions (Purcell 2010). Furthermore, consumption of sea cucumbers is thought to boost the immune system and to have aphrodisiac properties (Purcell 2010). Sea cucumbers may be sold live, fresh, or frozen in major consuming countries such as China, Hong Kong, Japan, South Korea, and Singapore (Toral-Granada et al. 2008; Purcell 2010). The frozen meat is often comprised of five longitudinal internal contractile muscle bands (Purcell 2010). More often, sea cucumbers are gutted, boiled, and dried to produce “*beche-de-mer*,” also referred to as “*trepang*” in Indonesia or “*hai-som*” in China (özer et al. 2004; Purcell 2010). When dried, “*beche-de me*r” is reconstituted by gentle boiling and subsequently consumed in sauces or soup dishes (Purcell 2010). In Japan and Korea, the body wall and viscera of sea cucumbers are also eaten raw, pickled, or fried (Çakli et al. 2004; özer et al. 2004).

During the past decade, the volume of scientific literature reporting on the biochemical properties of sea cucumber tissues grew significantly (Takashi et al. 2005). Sea cucumbers are rich in protein and low in fat (Wen et al. 2010), and are a natural source of mucopolysaccharides and chondroitin sulfate (Vieira and Mourão 1988; Kariya et al. 2004). Various harvested species also contain an assortment of bioactive molecules such as triterpene glycosides (Silchenko et al. 2005), some of which may be toxic at high concentrations (Alfonso et al. 2007). Regarding the protein fraction of sea cucumbers, Saito et al. (2002) demonstrated that the collagen fraction from *Parastichopus japonicus* body walls is rich in glutamic acid, an umami substance. Likewise, researchers have highlighted the antimicrobial properties of sea cucumber extracts (Villasin and Pomory 2000; Gowda et al. 2008; Cong et al. 2009). Mamelona et al. (2007) noted on the presence of flavonoids and phenols in *Cucumaria frondosa*, a sea cucumber species with sizable stocks in Atlantic Canada (Toral-Granada et al. 2008), and correlated these to antioxidant activity of extracts from digestive tract, muscle, and gonads. A more recent study compared in vitro antioxidant and antihypertensive activity of two differently processed tissues of *C. frondosa*, harvested from Icelandic waters, and it was demonstrated that these too were sources of bioactive ingredients (Hamaguchi et al. 2010).

Fresh or frozen longitudinal muscle bands are the more valued export product of the giant red sea cucumbers harvested in Alaska, and often the body wall is of considerably lower value. The body wall, dried and mostly marketed as “*beche-de-mer*,” is the largest fraction of *P. californicus* by weight, and the high concentration of glycosaminoglycans and collagen in the body wall suggests this may have significantly higher value if processed differently for nutraceutical or pharmaceutical applications (Liu et al. 2010). Even though numerous studies concerning the biochemistry of sea cucumbers have been published, there is little published information on *P. californicus* as a food. Liu et al. (2010) reported detailed information regarding the composition of the collagens of *P. californicus* tissues; nevertheless, the detailed fatty acid compositions of *P. californicus* body wall and longitudinal muscle bands have not been documented to date. Accordingly, the objective of this study was to determine the chemical composition and detailed fatty acid profiles of freeze-dried body wall and muscle of *P. californicus* commercially harvested in Alaska.

## Materials and Methods

### Sampling

Fifteen fresh *P. californicus* were harvested from Uyak Bay (Latitude ∼57° 26′ N, Longitude ∼153° 49′ W), Kodiak Island, Alaska, during the commercial dive fishery that occurs annually during October. Giant red sea cucumbers were delivered live, submerged in seawater at 8–9°C, to the University of Alaska's Kodiak Seafood and Marine Science Center pilot plant, and immediately processed. The body was slit lengthwise between ambulacra, drained of fluids, and weights recorded (4.4 kg total weight). The anterior end and viscera were removed, and the longitudinal and subjacent circular muscles separated from the body wall. The longitudinal muscles (1 kg) and body walls (3.4 kg) were separated in three groups of similar weight comprised of five sea cucumbers each. The six aggregate (composed of five individual *P. californicus*) samples (body walls *n *=* *3; muscle *n *=* *3) were individually frozen to −30°C in a walk-in freezer (Bally®; Morehead City, NC). Frozen samples were placed in a freeze drier (VirTis Virtual 52ES Freeze Dryer Lyophilizer; Gardiner, NY) and maintained at −30^°^C for 30 min then at −40^°^C for 30 min with a condenser temperature of −50^°^C and chamber pressure of 53.33 kPa. The primary freeze-drying parameters for shelf temperature and drying time were −40^°^C for 8 h, −30^°^C for 8 h, −20^°^C for 8 h, −10^°^C for 8 h, and 0^°^C for 8 h, all under 8 Pa. The secondary drying was set at 25^°^C for 6 h at 8 Pa. The freeze-drying process took 48 h. Freeze-dried samples were milled through a 2 mm mesh screen (Wiley Mill model ED5; Thomas Scientific, Swedesboro, NJ), and vacuum packed (Nylon/PE vacuum bags of 100 *μ*m film) using an UltraVac (Model UV2100-B; Koch, Kansas City, MO). Packaged samples were frozen at −40^°^C and chemical analyses were conducted within 90 days of obtaining the live animals.

### Analysis of proximate composition

Moisture content (method 952.08, Official Methods of Analysis of AOAC International 2005) was determined in duplicate for sea cucumber internal muscle bands (% wet weight) and their freeze-dried counterpart samples (% w/w). Protein (method 968.06, Official Methods of Analysis of AOAC International 2005), and ash (method 938.08, Official Methods of Analysis of AOAC International 2005) were determined in duplicate for freeze-dried sea cucumber body wall and muscle band samples (% w/w). Nitrogen content was accessed by pyrolysis, in duplicate for freeze-dried body wall and muscle band samples, as described in AOAC method 968.06 using a LECO FP-2000 nitrogen analyzer (LECO Co., St. Joseph, MO), and protein content was calculated as 6.25 times % N (Official Methods of Analysis of AOAC International 2005). Lipids were extracted, in duplicate, from sea cucumber freeze-dried body wall and muscle band aggregate samples, using the method of Folch et al. (1957). After lipid extraction, solvent was removed at 49^°^C on a rotary evaporator (Büchi Rotavapor R-205, Westbury, NY) and lipids transferred into a preweighed 10 mL amber screw-top vial. The remaining solvent was removed under a N_2_ gas stream until constant weight and percent lipids (% w/w) were determined. Oils were stored in chloroform containing 0.01% Butylhydroxyltoluene (BHT) at −70°C until analysis. Carbohydrate content (% w/w) of the six sea cucumber freeze-dried aggregate samples was determined, in duplicate, using a modification of the classic phenol sulfuric acid procedure of Dublois et al. (1956) with the standard curve constructed using purified glycogen.

### Analysis of fatty acids

Fatty acids methyl esters were prepared from each oil extract, obtained by the Folch et al. (1957) method, using 20 mg of lipids as described by Maxwell and Marmer (1983). A quantity of 1 mg of tricosanoic acid methyl ester (23:0) was used as an internal standard. Fatty acid methyl esters were quantified as previously described by Bechtel and Oliveira (2006). Data were collected and analyzed using the GC ChemStation program (Rev.A.08.03 [847]; Agilent Technologies 1990–2000, Wilmington, DE). All standards used in the identification of peaks were purchased from Supelco® (Bellefonte, PA). The standards used were Supelco 189-19, Bacterial Acid Methyl Esters Mix, Marine Oil #1, and Marine Oil #3.

### Analysis of amino acids and SDS-PAGE electrophoresis

Amino acid profiles were determined for each of the six freeze-dried sea cucumber aggregate samples by the AAA Service Laboratory Inc. (Boring, OR). Samples were hydrolyzed with 6*N* HCl and 2% phenol at 110°C for 22 h. Amino acids were quantified using the Beckman 6300 analyzer (Beckman-Coulter Inc., Brea, CA) with post column ninhydrin derivatization. Tryptophan and cysteine content were not determined.

The sodium dodecyl sulfate tricine/polyacrylamide gel electrophoresis system was used with a Photodyne Foto/Force 300 apparatus under reducing conditions according to Schagger and Von Jagow (1987). Precast 10–20% Tricine gels (Invitrogen Life Technologies, Carlsbad, CA) were used and molecular mass standards were purchased from Sigma-Aldrich. The protein bands were visualized from the gels stained with Coomassie blue (Sigma-Aldrich, St. Louis, MO) and molecular weights assigned (Bio-Rad Versa Doc 1000 Imaging System; Bio-Rad, Hercules, CA).

### Analysis of minerals

Mineral analyses were conducted in each of the six freeze-dried sea cucumber aggregate samples. Samples were digested using a wet ashing procedure, and element concentrations were measured using the Agilent 7500ce Inductively Coupled Plasma-Mass Spectrometer (ICP-MS) (Agilent Technologies Inc., Santa Clara, CA) housed in the Advanced Instrumentation Laboratory at the University of Alaska Fairbanks.

### Statistical analysis

Results were subjected to one-way analysis of variance, and reported weighted means and standard deviation of the means were determined using Statistica software v.8.0 (StatSoft Inc., Tulsa, OK). Tukey's Honest Significant Difference Test (*P* < 0.05) was used to determine significant differences in composition of sea cucumber body wall and muscle bands.

## Results and Discussion

### Proximate composition

The proximate composition of freeze-dried body wall and internal muscle bands of *P. californicus* is provided in Table 1. Overall, the results presented in this study are broadly similar to those found in previous investigations of *P. californicus* (Chang-Lee et al.^1989^; Liu et al. 2010) and for three other sea cucumber species, *Holothuria tremula* (Çakli et al. 2004), *Holothuria scabra* (özer et al. 2004), and *Cucumaria frondosa* (Zhong et al. 2007). Moisture content of *P. californicus* muscle bands was 84.5 ± 0.1% (wet weight) and this value is fairly similar to moisture content of 85.7 ± 0.3% (wet weight) reported by Liu et al. (2010) for *P. californicus* connective tissue compartment, which lays between the five longitudinal muscle bands and the integument. The connective tissue compartment is the body wall minus the outermost integument layer (similar to a vertebrate epidermis) that is only one cell thick. For the *P. californicus* body wall, also known as the skin, Liu et al. (2010) reported average moisture content of 90.1 ± 0.1% (wet weight), while Chang-Lee et al. (1989) reported a value of 88.8% (wet weight) for a *P. californicus* sample comprised of body wall, connective tissue, and muscle bands. Moisture content of *P. californicus* from Alaska waters is similar to values determined for another temperate–polar sea cucumber species, *Cucumaria frondosa*. Zhong et al. (2007) established that moisture contents of *C. frondosa* body wall and of *C. frondosa* body wall with internal organs are 87.4% and 90.1% (wet weight), respectively.

**Table 1:** Proximate composition (% w/w  ±  standard deviation) of freeze-dried *Parastichopus californicus* body wall and muscle bands

Composition	Body wall (N = 15; *n* = 3)	Muscle bands (N = 15; *n* = 3)
Protein	47.03 ± 0.53	68.40* ± 0.51
Ash	25.73* ± 0.25	12.18 ± 0.75
Carbohydrates	15.02* ±2.83	8.61 ± 0.38
Lipids	8.19* ± 0.27	5.30 ± 0.32
Moisture	4.03 ± 0.19	5.50* ± 0.60

* Denotes statistical significance between body wall and muscle bands at P < 0.05.

Proximate composition of freeze-dried body wall and muscle bands were markedly different (*P* < 0.05; Table 1). Freeze-dried muscle bands had significantly higher protein content and significantly lower ash, lipids, and carbohydrate contents than body wall. The most remarkable difference was in ash content of body wall, which was twice the value found in the muscle bands. This is most likely due to the presence of abundant microscopic skeletal elements, called ossicles, in sea cucumber body wall (Smiley 1994). Ossicles are formed within cellular vesicles and are composed of calcium carbonate. Wen et al. (2010) determined chemical composition and nutrition value of dried sea cucumber products produced from *Stichopus hermanni*, *Thelenota ananas*, *Thelenota anax*, *Holothuria fuscogilva*, *Holothuria fuscopunctata*, *Actinopyga mauritiana*, *Actinopyga caerulea*, and *Bohadschia argus*. These sea cucumber species are used for production of “*beche-de-mer*” in the Western Central Pacific region (Toral-Granada et al. 2008). Ash content of these commercial products, procured at a Chinese retail market, was highly variable ranging from 15.4% in *A. mauritiana* to 39.6% in *H. fuscopunctata* (Wen et al. 2010). Wen et al. (2010) also reported highly variable protein and lipid contents in these products, which ranged from 40.7% (*T. anax*) to 63.3% (*A. mauritiana*) and 0.3% (*H. fuscopunctata*) to 10.1% (*A. caerulea*), respectively. The *P. californicus* freeze-dried body wall and muscle bands have protein, lipid, and ash contents within these ranges (Table 1). Liu et al. (2010) reported average lipid content in wet weight of 0.57% and 0.44% for skin and connective tissue of giant red sea cucumber harvested in Alaska. Differences in lipid content of *P. californicus* body wall reported in our study as it compares to wet-weight data reported by Liu et al. (2010) may be attributed to presence of nonsaponifiable matter and/or nonlipid substances, such as pigments and complex carbohydrates, in the lipid extracts of the freeze-dried tissue. Nonlipid material, complex lipids, and lipid molecules associated to proteins in the form of lipoproteins may not be readily extractable from wet tissue; however, these compounds may coextract with polar and apolar lipids in freeze-dried tissue given the chloroform–methanol solvent system used. In a review of the physiologically active substances in sea cucumbers, Takashi et al. (2005) cited a diverse array of bioactive complex lipids such as ceramides and glycosphingolipids that have been recently isolated from lipid extracts of fresh or freeze-dried sea cucumber tissues. The elevated lipid contents observed in Table 1 indicate that further research is needed to investigate the complete chemical makeup of lipid extracts obtained from freeze-dried products of *P. californicus* by the method of Folch et al. (1957).

The carbohydrate assay values were highest for the freeze-dried body wall at 15%, nearly twice the concentration found in muscle (Table 1). Liu et al. (2010) did not report carbohydrate content of *P. californicus*, and carbohydrate contents reported in Table 1 are much higher than previously reported by Chang-Lee et al. (1989) for eviscerated uncooked (0.2% wet w/w) and cooked-dried *P. californicus* (2.7% dry w/w). These differences may be attributed to the fact that in this study, sea cucumbers were received live and immediately processed into freeze-dried products, which reduces possibility of chemical degradation of tissues, instead of being processed 24 h post-mortem and dried in a gravity-convection oven at 60°C for 36 h (Chang-Lee et al. 1989). Iozzo (1998) noted that high concentrations of amino sugars or glycosaminoglycans were present in sea cucumber connective tissue, while Alfonso et al. (2007) surmised that the high natural proteoglycan levels of sea cucumber body wall might make this tissue of interest to the nutraceutical industry. Similarly, the 15% (w/w) carbohydrate content recorded for *P. californicus* freeze-dried body wall (Table 1) indicate that further studies should be conducted to elucidate the detailed composition of carbohydrates present in this product, as it has been done for *P. japonicus* (Kariya et al. 2004).

### Fatty acid profiles

The detailed fatty acid composition of the lipids extracted from the freeze-dried body wall and longitudinal muscle bands of *P. californicus* is given in Table 2. Statistical analysis was conducted for only those fatty acids whose abundance exceeded 2%. A majority of fatty acids were identified in both freeze-dried tissues (∼95%); however, 5% of peaks present in the chromatograms represented unknowns. Fatty acids below the detection limit of integration (0.015%) were not investigated further. Drazen et al. (2008) noted a number of unusual fatty acids in abyssal Echinoderms such as two nonmethylene interrupted diunsaturated fatty acids (22:2NMI), and two fatty acids containing hydroxyl groups (αOH23:1; αOH-24:1). The fatty acid 23:1*n*-9, previously identified in Holothuroidea by Kaneniwa et al. (1986), was later confirmed in dried sea cucumbers by Kasai (2003) and Wen et al. (2010) in eight different sea cucumbers species. This fatty acid could not be positively identified in this study. Kaneniwa et al. (1986) showed that this unusual fatty acid eluted in his gas chromatographer system after 23:0 and before 22:5*n*-3. An unknown fatty acid peak with abundance of 3–4% and retention time of 41.50 min, eluting 0.2 min after the internal standard peak (23:0; 40.30 min), may well correspond to 23:1*n*-9. However, this fatty acid is not commonly found in marine oil extracts and a commercial standard was not available. Additional research, using a gas chromatography coupled to a mass spectrometer, is needed to reveal the identity of this particular fatty acid peak in *P. californicus*.

**Table 2:** Fatty acid composition (% total fatty acids ± standard deviation) of freeze-dried *Parastichopus californicus* body wall and muscle bands.

FA**	Body wall (*N* = 15; *n* = 3)	Muscle bands (*N* = 15; *n* = 3)
*anteiso-14:0*	0.88 ± 0.06	0.53 ± 0.02
14:0	1.82 ± 0.04	1.13 ± 0.01
*iso*-15:0	0.25 ± 0.00	0.20 ± 0.00
*anteiso-*15:0	1.45 ± 0.03	0.61 ± 0.53
16:0	10.90* ± 0.09	6.01 ± 0.06
*iso*-17:0	0.87 ± 0.15	0.50 ± 0.04
*anteiso-*17:0	0.44 ± 0.25	0.43 ± 0.01
17:0	0.95 ± 0.02	0.69 ± 0.01
18:0	6.84* ± 0.05	5.70 ± 0.03
20:0	1.30 ± 0.01	1.13 ± 0.01
22:0	0.60 ± 0.01	BDL
24:0	0.64 ± 0.32	1.21 ± 0.01
∑ *Saturated FA*	26.94* ± *0.14*	*18.13* ± *0.21*
16:1*n*-11	0.87 ± 0.15	0.50 ± 0.04
16:1*n*-9	1.07 ± 0.08	0.56 ± 0.01
16:1*n*-7	14.99* ± 0.06	8.57 ± 0.08
16:1*n*-5	1.01 ± 0.01	0.58 ± 0.01
18:1*n*-9 *cis*	4.96* ± 0.10	2.86 ± 0.02
18:1*n*-7	5.60 ± 0.03^b^	5.80* ± 0.05
18:1*n*-5	0.48 ± 0.01	0.50 ± 0.01
20:1*n*-11	2.56 ± 0.06^b^	3.84* ± 0.04
20:1*n*-9	2.49* ± 0.19^a^	1.59 ± 0.02
20:1*n*-7	1.01 ± 0.15^a^	1.09 ± 0.05
22:1*n*-9	1.30 ± 0.02	0.96 ± 0.02
22:1*n*-7	1.79 ± 0.03	3.60* ± 0.09
24:1*n*-9	1.67 ± 0.01	1.60 ± 0.05
∑ *Monounsaturated FA*	39.80* ± *0.16*	*32.05* ± *0.54*
18:2*n*-6 *cis*	1.04 ± 0.07	0.64 ± 0.01
18:3*n*-6	1.37 ± 0.02	0.42 ± 0.37
20:2*n*-6	0.73 ± 0.03	1.12 ± 0.01
20:4*n*-6 (ARA)	7.05 ± 0.03	9.90* ± 0.17
20:4*n*-3	0.16 ± 0.01	BDL
20:5*n*-3 (EPA)	12.34 ± 0.04	22.63* ± 0.48
22:6*n*-3 (DHA)	6.19 ± 0.06	8.93* ± 0.13
∑ *Polyunsaturated FA*	*28.88* ± *0.06*	43.64* ± *0.75*
∑ *n*-3	18.69 ± 0.09	31.56* ± 0.61
∑ *n*-6	10.19 ± 0.08	12.08* ± 0.41
Ratio *n*-3: *n*-6	1.83 ± 0.03	2.61* ± 0.12

* Denotes statistical significance between body wall and muscle bands at P < 0.05.

** Statistical analysis conducted only for FA > 2% w/w; BDL, below detection limit = 0.015%; FA, fatty acids.

There were marked differences between the fatty acid profiles of the body wall and muscle band samples. Monounsaturated fatty acids were the most abundant class of fatty acids in freeze-dried body wall, while polyunsaturated fatty acids were the predominant fatty acid class in freeze-dried muscle bands. The predominant monounsaturated fatty acid in both tissues was palmitoleic acid (16:1*n*-7), and this was also reported to be the case for *Cucumaria frondosa* (Zhong et al. 2007) and for Cucumaria sp. (Kaneniwa et al. 1986). The monounsaturated fatty acids in body wall and muscle bands of *P. californicus* were significantly higher than values reported for holothurians from tropical and temperate waters (Svetashev et al. 1991), but within the range (27–46% total fatty acids) observed for eight dried products produced from sea cucumber species of the Western Central Pacific region (Wen et al. 2010). The content of monounsaturated fatty acids of four echinoderm species of the North-East Pacific Ocean ranged from 28% to 45% of total fatty acids, with most pronounced differences occurring in the content of 20:1*n*-11 (Drazen et al. 2008). Likewise, a significant difference in the abundance of 20:1*n*-11 was observed between body wall and muscle bands. Presence of substantial concentrations of 20:1 and 22:1 fatty acids in the lipids of marine organisms of northern latitudes have been previously recorded (Ackman 1989a). The origin of these fatty acids in cold-water fish species has been attributed to copepods (Ackman et al. 1980; Ackman 1989b). Sea cucumbers are detritivorous species and extract nutrients from sediment, but some species also feed on plankton entrapped by tentacles surrounding the mouth (Ackman 1989a). The various 20:1 and 22:1 fatty acid isomers detected in the body wall and muscle bands of *P. californicus* likely originate from their diet.

The large concentrations of polyunsaturated fatty acids recorded for *P. californicus* muscle bands are consistent with reports by Svetashev et al. (1991) for shallow-water holothurians. The most abundant polyunsaturated fatty acids in *P. californicus* (Table 2) were eicosapentaenoic acid (EPA; 20:5*n*-3) and arachidonic acid (ARA; 20:4*n*-6). This is in agreement with observation by Ackman (1989a), who indicated that these fatty acids are prominent in holonturians. It has been established that cold-water marine species tend to accumulate higher amounts of long-chain *n*-3 fatty acids, particularly EPA and DHA (docosahexaenoic acid; 22:6*n*-3), than species found in temperate climate (Valentine and Valentine 2010). Environmental temperature affects fatty acid composition in marine organisms, and a suspected driving force for accumulation of long-chain *n*-3 fatty acids is their ability to sustain membrane fluidity at lower temperatures (Valentine and Valentine 2010). The lipid bilayers of cellular membranes are rich in long-chain *n*-3 fatty acids because they confer antifreeze properties, allowing cells to operate at very low temperatures (Valentine and Valentine 2010). Long-chain *n*-3 fatty acids are also of considerable interest to human nutrition as they play a crucial role in brain function as well as normal growth and development. These fatty acids also may reduce the risk of coronary heart disease, cancer, inflammation, and arthritis (Ruxton et al. 2005). Long-chain *n*-3 fatty acids are highly concentrated in the human brain and appear to be important for cognitive (brain memory and performance), and behavioral function (Valentine and Valentine 2010). In this study, *P. californicus* muscle bands had higher concentrations of both EPA and DHA than that of body wall. Similar results relating to higher concentration of EPA over DHA in deep-sea echinoderms were reported by Drazen et al. (2008), who documented ranges of 9–18% total fatty acids of EPA and 1–8% total fatty acids of DHA. Zhong et al. (2007) reported very high EPA content in *C. frondosa* from Newfoundland (Canada), with levels ranging from 43% to 57% (w/w), and highlighted the role this fatty acid has in blood clotting because it possesses antithrombotic activity. In *C. frondosa*, DHA content ranged from 2% to 6% (Zhong et al. 2007). In a separate study of the fatty acids in *Cucumaria* sp., Kaneniwa et al. (1986) reported EPA and DHA content of 36.9% and 1.1% (w/w), respectively. For eight dried products derived from sea cucumbers of the Western Central Pacific, Wen et al. (2010) found EPA content ranging from 0.3% to 3.9% total fatty acids. Interestingly, the authors reported that DHA was not detected in any of the dried sea cucumber products investigated (Wen et al. 2010). Conversely, Fredalina et al. (1999) determined the fatty acid profile of crude extracts made from *Stichopus chloronotus*, and reported EPA and DHA contents similar to those given in Table 2.

Of particular interest is the arachidonic acid (ARA) content in *P. californicus* (Table 2), which was the principal *n*-6 polyunsaturated fatty acid detected. ARA plays an important role in human health, especially in growth because it is the main precursor of eicosanoids (Gil 2002), the major component of cell membrane phospholipids. ARA is also an important constituent of the central nervous system (Carlson and Neuringer 1999). Wen et al. (2010) reported a wide range of ARA content (1.8–14.4% total fatty acids) in dried sea cucumber products, whereas values above 20% were reported for tropical sea cucumber species harvested in Vietnamese waters (Svetashev et al. 1991). The ARA contents in Table 2 are similar to those reported by Drazen et al. (2008) for the following abyssal sea cucumbers*, Abyssocucumis abyssorum* (6.8% total fatty acids), *Peniagone vitrea* (8.5% total fatty acids), and *Protankyra brychia* (12.1% total fatty acids). Interestingly, the ARA content of sea cucumbers appears to be consistently higher than those found in marine fish.

Palmitic acid (16:0) was the major saturated fatty acid in the *P. californicus* body wall, being significantly lower in the muscle bands. Stearic acid (18:0) content in muscle bands was similar to the content of 16:0, but body wall had much lower content of 18:0 than that of 16:0. All other saturated fatty acids were detected at concentrations below 2%. In *Cucumaria frondosa*, 16:0 and 18:0 ranged from 2.1% to 2.8% (w/w) and from 1.5% to 4.2% (w/w), respectively. While in Wen et al. (2010) study, 16:0 and 18:0 contents were much higher, ranging from 5.9 to 31.8% (total fatty acids) and from 7.1% to 14.1% (total fatty acids), respectively. For *P. japonicus*, the contents of 16:0 and 18:0 seem to fluctuate with season (Kaneniwa et al. 1986). Specimen collected in Japanese waters during May of 1979 and October of 1985 had 16:0 and 18:0 contents of 8.2% and 10.8%, and of 16.0% and 6.7%, respectively. Takashi et al. (2005) discussed the presence of 12-methyltetradecanoic acid, also named anteiso 15:0, in the lipids of *C. frondosa* in light of its antiproliferative effects on prostate cancer cells. Anteiso 15:0 was detected in the lipids extracted from freeze-dried body wall and muscle bands of *P. californicus*, but at concentrations below 1.5% (Table 2). Furthermore, several other branched saturated fatty acids were present in the freeze-dried *P. californicus* tissues studied, and this is consistent with results reported for numerous sea cucumbers species (Kaneniwa et al. 1986; Svetashev et al. 1991; Kasai 2003; Zhong et al. 2007; Drazen et al. 2008).

This is, to the best of our knowledge, the first study documenting the fatty acid composition of *P. californicus*. Overall, the fatty acid profiles of body wall and muscle bands of this sea cucumber resemble those described for other species; however, the distribution and occurrence of certain fatty acids is unique to this species and representative of the fatty acid composition of temperate–polar marine organisms.

### Amino acid profiles

Table 3 lists the amino acid composition of *P. californicus* as a percent of total amino acid composition on a weight basis. Significant differences between freeze-dried muscle bands and body wall (*P* < 0.05) were found for all amino acids; however, the most abundant amino acids for both tissues are glutamic acid, aspartic acids, and arginine. This is consistent with data reported by Wen et al. (2010) for eight dried sea cucumber species. Liu et al. (2010) and Saito et al. (2002) have determined the detailed amino acid composition of pepsin-solubilized collagen (PSC) of *P. californicus* and *P. japonicus*, respectively. Liu et al. (2010) observed that the PSC of body wall and connective tissue of *P. californicus* were similar to those of the congeneric *P. japonicus*, being rich in glycine, glutamic acid, alanine, proline, and aspartic acid. Furthermore, the authors observed that *P. californicus* body wall contained higher amounts of glycine, hydroxyproline, and proline than that of connective tissue; conversely, skin had lower contents of leucine and phenyalanine (Liu et al. 2010). Their findings are well aligned to data shown in Table 4 for body wall and muscle bands, and this demonstrates that amino acid composition of *P. californicus* muscle bands resemble that of its connective tissue. Liu et al. (2010) have demonstrated that *P. californicus* tissues have type I collagens of good gel-forming ability at pH 6.5; although, their peptide maps differs from calfskin type I collagen. This particular characteristic of the collagen fraction of *P. californicus* indicates that it may serve as a marine collagen alternative to mammalian collagen.

**Table 3:** Amino acid composition (% total amino acids ± standard deviation) of freeze-dried Parastichopus californicus body wall and muscle bands.

Amino acid	Body wall (*N* = 15; *n* = 3)	Muscle bands (*N* = 15; *n* = 3)
Alanine	5.6* ± 0.1	5.0 ± 0.0
Arginine	8.4 ± 0.1	13.4* ± 0.1
Aspartic acid	11.8* ± 0.0	10.9 ± 0.1
Glutamic acid	13.1 ± 0.2	16.1* ± 0.0
Glycine	12.3* ± 0.2	4.8 ± 0.1
Histidine	1.7 ± 0.0	2.1* ± 0.0
Hydroxyproline	2.6* ± 0.0	0.3 ± 0.0
Isoleucine	3.6 ± 0.0	4.2* ± 0.0
Leucine	5.3 ± 0.0	7.6* ± 0.0
Lysine	4.1 ± 0.1	7.6* ± 0.0
Methionine	2.2 ± 0.1	2.3* ± 0.0
Phenylalanine	3.8 ± 0.0	4.1* ± 0.0
Proline	6.7* ± 0.1	3.8 ± 0.0
Serine	5.1* ± 0.1	4.4 ± 0.0
Threonine	5.7* ± 0.0	4.6 ± 0.1
Tyrosine	3.4 ± 0.0	3.5* ± 0.0
Valine	4.8 ± 0.0	5.3* ± 0.0

* Denotes statistical significance between body wall and muscle bands at P < 0.05.

Of particular note is the high amount of glycine (12%) detected in *P. californicus* body wall compared with the muscle bands (4.8%). Wen et al. (2010) also documented high content of glycine in dried sea cucumbers, which ranged from 5.77 to 12.5 g/100 g^−1^ wet weight corresponding to 126–216 mg/g crude protein. It has been shown that glycine reduces levels of total cholesterol in serum (Wen et al. 2010), and its abundance in *P. californicus* freeze-dried body wall should be noted.

In muscle bands of *P. californicus,* there are high amounts of the basic amino acid lysine and very high contents of arginine. The ratio of lysine to arginine in muscle bands and body wall are 0.57% and 0.48%, respectively. Previous reports suggested that low lysine-to-arginine ratio significantly reduces serum cholesterol (Sugano et al. 1984; Rajamohan and Kurup 1997). Our results concur with those of Wen et al. (2010), who observed that sea cucumbers have much lower lysine-to-arginine ratios than those found in many other seafood products including fish (Zuraini et al. 2006; Zhao et al. 2010), crab (Naczk et al. 2004), and shrimp (Inhamuns et al. 2009). The values of essential amino acids, except threonine, were significantly higher in muscle bands than in body wall of *P. californicus* (Table 3). Bingham (1977) stated that the essential amino acids most often limiting are lysine, methionine, threonine, and tryptophan. In this study, histidine and methionine were the limiting amino acids, while Wen et al. (2010) found the three limiting essential amino acids in the eight dried sea cucumber products investigated to be histidine, lysine, and methionine.

The electrophoretic profiles for *P. californicus* proteins from both body wall and muscle were determined (SDS-PAGE gel not shown). The muscle tissue had several bands consistent with the banding pattern seen in muscle tissues including myosin at 200 kDa and actin at 44 kDa. The body wall had fewer protein bands and only one major band with apparent molecular weight of 183 kDa. Although the identification of this band was not made, it is interesting to see that body wall tissue had such a high content of a single molecular weight band.

### Mineral profiles

Information regarding mineral composition of sea cucumber products is scant, and one of the only studies identified was by Chang-Lee et al. (1989), who reported mineral content of eviscerated uncooked and dried *P. californicus* and the congeneric *P. parvimensis*, harvested in California. Table 4 shows the mineral composition of both body wall and muscle bands of *P. californicus*, and significant differences were detected in the contents of all macro- and microelements. The concentration of calcium in the body wall was 10-fold higher than that of muscle bands. This is due to presence of cellular vesicles in the body wall, which contain calcium carbonate (Smiley 1994). Chang-Lee et al. (1989) pointed out that calcium has been reported to be present in sea cucumbers in large quantities. Overall, Chang-Lee et al. (1989) results for some of the macroelements quantified in sea cucumbers differ substantially from data shown in Table 4, while concentrations of most microelements are comparable. For instance, Chang-Lee et al. (1989) reported much lower concentrations of sodium and magnesium in oven-dried *P. californicus* and *P. parvimensis* at 39 and 132 mg/100 g, and at 58 and 31 mg/100 g, respectively. Furthermore, reported concentrations of phosphorous, potassium, and sodium in these oven-dried products were also lower at 100–120 mg/100 g of product, 208–286 mg/100 g of product, and 652–668 mg/100 g of product, respectively (Chang-Lee et al. 1989). On the other hand, Chang-Lee et al. (1989) findings for copper and zinc contents were in the ranges of 4.2–6.6 ppm and 38.2–25.2 ppm, respectively, which are similar to values given in Table 4 for body wall. Xing and Chia (1997) determined that concentration of copper (0.25 ppm dry weight) in the longitudinal muscle bands of *Holonthuria leucospilota*, the large black sea cucumber, is much lower than zinc (97.27 ppm dry weight) and that zinc concentrations were 10-fold higher in muscle bands than in body wall (8.14 ppm dry weight). These observations are well aligned to data shown in Table 4. Interestingly, the authors pointed out that *H. leucospilota* has unusually high zinc and rather low copper concentrations in their muscle bands, when compared with most mollusks and crustaceans (Xing and Chia 1997), and suggested that the muscle tissue of the large black sea cucumber is unique with regards to its mineral profile. Iron content of 668 ppm determined in oven dried eviscerated *P. californicus* was much higher than the 134 ppm found in oven-dried *P. parvimensis (*Chang-Lee et al. 1989*);* the latter was comparable to the 184 ppm of iron detected in *P. californicus* body wall (Table 4). In summary, longitudinal muscle bands had a mineral complement characteristic of seafood, but with high levels of zinc, and this observation is in close agreement to previous findings for another sea cucumber species (Xing and Chia 1997).

**Table 4:** Mineral composition (average ± standard deviation) of freeze-dried *Parastichopus californicus* body wall and muscle bands.

Units	Minerals	Body wall (N = 15; n *=* 3)	Muscle bands (N = 15; n = 3)
	Ca	2.5*± 0.2	0.2 ± 0.0
	K	0.4 ± 0.1	1.0* ± 0.1
% w/w	Mg	1.4* ± 0.0	0.5 ± 0.0
	Na	8.8* ± 0.2	4.5 ± 0.1
	P	0.5 ± 0.0	1.6* ± 0.0

	Cr	6.7* ± 2.4	1.0 ± 0.0
	Cu	3.5* ± 0.2	2.2 ± 0.2
	Fe	184.2* ± 151.4	24.6 ± 2.1
mg/kg	Li	6.6* ± 0.6	3.5 ± 0.4
	Mn	43.6* ± 4.3	0.9 ± 0.0
	Ni	4.0* ± 1.3	2.8 ± 4.2
	Zn	40.4 ± 4.3	128.5* ± 2.9

* Denotes statistical significance between body wall and muscle bands at P < 0.05.

## Conclusions

The giant red sea cucumber, *Parastichopus californicus*, is the only holothurian species harvested in Alaska. Fresh or frozen longitudinal muscle bands are the principal export product. The body wall, mostly dried and marketed as “*beche-de-mer,*” is the largest tissue of *P. californicus* and has much lower market value than the muscle. In this initial chemical characterization of *P. californicus* products, freeze-dried samples were exploited. Our analyses showed that muscle bands contained a considerable amount of high-quality marine protein, while the body wall had a greater percent of lipids. The concentration of protein found in the body wall was lower than muscle, partly due to its higher ash content. Analysis showed that longitudinal muscle bands had a mineral complement characteristic of seafood, but with high levels of zinc. Amino acid composition indicated that much of the body wall protein was similar in many respects to connective tissue protein. PAGE electrophoresis in the presence of SDS identified one major protein band in the body wall with an apparent molecular weight of 183 kDa.

This is, to the best of our knowledge, the first study documenting the fatty acid composition of *P. californicus*. Muscle bands had a higher proportion of the nutritionally important long-chain *n*-3 fatty acids, and exceptionally high content of eicosapentaenoic acid (20:5*n*-3) was observed. High content arachidonic acid (20:4*n*-6), a physiologically active long-chain *n*-6 fatty acid, was also recorded. Overall, the fatty acid profiles of body wall and muscle bands of *P. californicus* resemble those described for other sea cucumber species; however, the distribution and occurrence of certain fatty acids is unique to this species and representative of the fatty acid composition of temperate–polar marine organisms. The chemical characterization of freeze-dried body wall from the giant red sea cucumber demonstrated that this species has unusual and valuable nutritional properties, and if appropriately processed, could be better utilized for nutraceuticals and pharmaceutical applications.
